# Gut Microbiota: An Important Participant in Childhood Obesity

**DOI:** 10.1016/j.advnut.2024.100362

**Published:** 2024-12-28

**Authors:** Yu Luo, Maojun Li, Dan Luo, Binzhi Tang

**Affiliations:** 1 Department of Pediatrics, Sichuan Provincial People’s Hospital, School of Medicine, University of Electronic Science and Technology of China, Chengdu, China; 2 Department of Pediatrics, School of Medicine and Life Science of Chengdu University of Traditional Chinese Medicine, Chengdu, China

**Keywords:** gut microbiota, children, obesity, influence factors, mechanisms, treatment

## Abstract

Increasing prevalence of childhood obesity has emerged as a critical global public health concern. Recent studies have challenged the previous belief that obesity was solely a result of excessive caloric intake. Alterations in early-life gut microbiota can contribute to childhood obesity through their influence on nutrient absorption and metabolism, initiation of inflammatory responses, and regulation of gut–brain communication. The gut microbiota is increasingly acknowledged to play a crucial role in human health, as certain beneficial bacteria have been scientifically proven to possess the capacity to reduce body fat content and enhance intestinal barrier function and their metabolic products to exhibit anti-inflammatory effect. Examples of such microbes include bifidobacteria, *Akkermansia muciniphila*, and *Lactobacillus reuteri*. In contrast, an increase in Enterobacteriaceae and propionate-producing bacteria (Prevotellaceae and Veillonellaceae) has been implicated in the induction of low-grade systemic inflammation and disturbances in lipid metabolism, which can predispose individuals to obesity. Studies have demonstrated that modulating the gut microbiota through diet, lifestyle changes, prebiotics, probiotics, or fecal microbiota transplantation may contribute to gut homeostasis and the management of obesity and its associated comorbidities. This review aimed to elucidate the impact of alterations in gut microbiota composition during early life on childhood obesity and explores the mechanisms by which gut microbiota contributes to the pathogenesis of obesity and specifically focused on recent advances in using short-chain fatty acids for regulating gut microbiota and ameliorating obesity. Additionally, it aimed to discuss the therapeutic strategies for childhood obesity from the perspective of gut microbiota, aiming to provide a theoretical foundation for interventions targeting pediatric obesity based on gut microbiota.

**Statement of Significance**

We provide a summary of the factors, mechanisms, and therapeutic strategies pertaining to the impact of gut microbiota alterations on childhood obesity, with particularly emphasis recent advancements in leveraging short-chain fatty acids for modulating gut microbiota composition and ameliorating obesity-related concerns.

## Introduction

According to data from the WHO and World Obesity Federation, nearly 340 million (over 18%) of children and adolescents aged 5∼19 y are currently overweight or obese globally, and the figure continues to grow. The prevalence of obesity among children and adolescents aged 5 to 19 y worldwide witnessed an almost 10-fold increase over the past 4 decades. Obviously, the prevalence of obesity has reached epidemic levels [1,2]. Once considered a problem in high-income countries, overweight and obesity are now on the rise in low- and middle-income countries. In 2019, nearly half of all children under the age of 5 who were overweight or obese live in Asia [3]. Obesity-induced metabolism disorders not only impair the normal growth and development of children but also increase the incidence and mortality of type 2 diabetes mellitus (T2DM), cardiovascular disease, nonalcoholic fatty liver disease (NAFLD), and certain cancers such as breast cancer and liver cancer [4]. Statistics showed that ≤90% of individuals who experienced obesity during childhood or adolescence were likely to persist with obesity into adulthood. The rapid rise in childhood obesity has emerged as a pivotal underlying factor contributing to the surge in adult obesity [5,6].

Multiple studies have consistently demonstrated that alterations in the composition, abundance, and relative distribution of gut microbiota in pediatric individuals are affected by obesity, which influences the energy metabolism pathway from various aspects such as genetics, intrauterine microbiota exposure, mode of delivery, and geographic factors. The comparison of gut microbiota between children with obesity and those with normal weight revealed a significantly higher abundance of Firmicutes and a lower abundance of Bacteroidetes, and following dietary control, there was an observed reversal in the proportions of these 2 phyla, resulting in weight loss. The alteration in the proportion of intestinal Bacteroidetes and Firmicutes could potentially be linked to childhood obesity [[7], [8], [9]]. Recent findings have demonstrated that an increase in the abundance of specific Firmicutes and Bacteroidetes species is associated with obesity and intestinal inflammation, respectively [10,11]. The dysbiosis of the gut microbiota may be a crucial factor contributing to childhood obesity. Current studies have revealed that therapeutic strategies targeting the gut microbiota exhibit a discernible ameliorative effect on childhood obesity. The management of obesity through dietary intervention, probiotic supplementation, and fecal microbiota transplantation (FMT) are prominent areas of investigation [12]. However, the substantial interindividual variations in gut microbiota composition and the inherent variability in microbiota composition, gene expression, and function within an individual further compound the aforementioned intricacy, thereby augmenting the challenges associated with elucidating the intricate relationship between gut microbiota and childhood obesity as well as its underlying mechanism of action [13]. Therefore, this article aimed to provide comprehensive insights into managing childhood obesity by reviewing recent advancements in this field with a specific emphasis on targeting the gut microbiota.

## Intestinal Microecology and Childhood Obesity

Recent findings have demonstrated a strong correlation between the progression of obesity and alterations in gut microecology [14]. The intestinal tract harbors the most dominant and intricate microecosystem of the human body, comprising intestinal tissues, fungi, Archaea, viruses, protozoans, as well as ∼100 trillion bacteria and their metabolites that constitute the gut microbiota. Through the Human Microbiome Project conducted by the NIH, 2172 species have been isolated from the human intestine. Among them, 386 are classified as obligate anaerobes, and the detected bacteria can be categorized into 12 phyla, with Firmicutes, Bacteroidetes, Actinobacteria, and Proteobacteria, accounting for a combined total of 93.5% [15]. The gut microbiota plays a crucial role in maintaining various physiologic homeostatic processes within the host, including the fermentation of indigestible dietary compounds, involvement in cholesterol and bile acid metabolism, as well as indirect regulation of mood and social behavior through the gut–brain axis (GBA). While disruption of this delicate balance due to alterations in either the quantity or composition of gut microbiota can lead to a spectrum of diseases such as inflammatory bowel disease (IBD) and gastrointestinal cancers [16].

Bacteriophages, as a crucial component of the gut microbiota, establish colonization within the intestines from the early stages of life. In a healthy human gut, only a small fraction of prophages was induced and activated to become independent extracellular phages. Nevertheless, alterations in the surrounding environment such as dietary changes, antibiotic usage, and inflammatory status can stimulate the activation or increase in lytic phage proportion within the intestinal prophage population, leading to an imbalance in phage ratios [17]. CrAssphage is a prevalent and abundant family in the gut virome across human populations, comprising ∼90% of the gut virome sequence [18]. Research findings indicated that in healthy children with normal weight, the abundance of the crAssphage Alpha subfamily is higher compared with individuals who are obese and with metabolic syndrome (MS). In individuals with obesity and MS, this difference becomes more pronounced due to decreased stability of the crAssphage Alpha subfamily and increased in the number of Delta subfamily [19]. Additionly, the gut virome of children with obesity undergoes changes alongside alterations in the microbiome. A study demonstrated that a positive correlation between abundance of phage contig 2740 and *Collinsella aerofaciens*, which is significantly overabundant in the group with obesity and MS [20]. Conversely, the phage contig 313 presented a negative correlation with the genus *Phascolarctobacterium*. Researchers speculated that the increased abundance of this phage contig in individuals with obesity may inhibit the abundance of *Phascolarctobacterium*, a potential protective bacterium against obesity [21]. Furthermore, increased abundance of phage contigs 207 and 540 in the healthy children with normal weight group associated with decreased abundance of Erysipelotrichaceae. This suggests that phage contigs 207 and 540 could be used to reduce Erysipelotrichaceae concentrations in groups affected by obesity or MS [20]. These findings provide possibilities for using bacteriophages as a therapeutic option against obesity-related changes in gut microbiota. In early stages, manipulation of the gut microbiota using bacteriophages may offer potential for preventing and treating obesity-related alterations in gut microbiota. In the future, it is promising to use bacteriophages for FMT in treating obesity and MS.

Additionally, as integral constituents of the gut microbiota, intestinal fungi also play a pivotal role in host metabolism and early-stage development of childhood obesity. Researchers investigated the association between gut microbiota and body mass index *z*-scores (BMIz) within the first 5 y after birth in a cohort of 100 infants from the Canadian Healthy Infant Longitudinal Development study. The findings revealed that an increase in fungal diversity during the initial year postpartum was linked to BMI values of both parents and infants, while the relationship between diversity patterns and early-life BMIz was influenced by maternal BMI, maternal dietary habits, infant antibiotic exposure, and bacterial β-diversity. Furthermore, variations in abundance of *Saccharomyces*, *Rhodotorula*, and *Malassezia* species exhibited distinct associations with early-life BMIz [22]. Moreover, a study conducted by Mercer et al. [23] revealed an inclination toward increased atypical α diversity in intestinal fungi among infants at the age of 3 mo. Notably, there was an enrichment of *Candida* species and a reduction in the abundance of *Malassezia* and *Cladosporium* species. These findings highlighted a clear association between fungal composition maturity and infant growth trajectory, emphasizing the necessity for incorporating fungi into broader-scale microbiome in pediatric research. Animal models have demonstrated the potential therapeutic role of *Candida bracarensis* in managing obesity and T2DM [24], and a study involving children revealed a correlation between obesity and decreased concentrations of *Candida* spp and *Saccharomyces* spp [25]. Subsequently, an experiment by orally administered *Candida parapsilosis* to mice induced with a high-fat diet (HFD), substantiated a causal relationship between the proliferation of *C parapsilosis* and diet-induced obesity, highlighting its significance [26]. Currently, there is a lack of research on the association between gut fungal colonization in infancy and obesity. Considering the role of infant gut fungi and gut bacteria in gut microbiota homeostasis and immunoregulation, they are intricately linked to the development of intestinal diseases, metabolic disorders, and allergic conditions. Further investigations are needed to elucidate the establishment and evolution process of gut fungi as well as their intricate relationship with both health and disease.

In comparison with adults, the gut microbiota of children under the age of 3 y exhibits reduced diversity and increased variability. The gut microbiota diversity was found to be reduced and the bacterial community structure exhibited alterations in children with obesity, as compared with their normal weight counterparts. By collecting fecal samples from children with normal weight and children with obesity, the results of 16S rDNA sequencing revealed a decreased α-diversity in the obesity group. Additionally, the genus *Prevotella* and the phylum Firmicutes exhibited higher abundance in the group with obesity, while *Sanguibacteroides* and *Bacteroides* were more prevalent in the control group [27]. It has been identified that *Escherichia coli* and *Shigella* spp are potentially associated with inflammation-dependent obesity and insulin resistance, and the genus *Lactobacillus* is linked to a higher BMI, while the genus *Staphylococcus* is associated with a lower BMI [28]. Another study conducted by Riva et al. [9] revealed an increase in the abundance of *Ruminococcus* sp. and, meanwhile, a decline in concentrations of bifidobacteria and *Akkermansia muciniphila* within the intestinal tract of children experiencing obesity, suggesting that the presence of *A muciniphila* in humans gut was associated with leanness. At the genus level, there was a significant increase in the relative abundance of *Faecalibacterium*, *Phascolarctobacterium*, and *Lachnospira* within the gut microbiota in individuals with obesity. Conversely, there was a notable decrease in the relative abundance of *Oscillospira* and *Dialister* species [29,30]. An early study revealed a significant increase in the abundance of *Faecalibacterium prausnitzii,* a butyrate-producing and anti-inflammatory bacterium from the family *Ruminococcaceae*, in children with obesity compared with that in nonobese children [31]. However, current research predominantly suggested a decreased abundance of *F prausnitzii* in adults with obesity [32]. Considering that butyrate can regulate mitochondrial fatty acid β-oxidation through epigenetic mechanisms to prevent HFD-induced insulin insensitivity and improve obesity-related characteristics, the utilization of *F prausnitzii* or its derivatives may present a promising alternative for the treatment of intestinal diseases associated with obesity and its complications. It has been previously revealed that *Phascolarctobacterium* possesses the capability to synthesize short-chain fatty acids (SCFAs) such as acetate and propionate, which are closely associated with enhancing host metabolism and regulating mood [33]. Subsequently, a study conducted by researchers from the Mayo Clinic identified elevated concentrations of *Phascolarctobacterium* in individuals who demonstrated a heightened inclination toward weight loss [34]. Furthermore, the genus *Lachnospira*, which belongs to the phylum Firmicutes, is commonly found in the intestines of healthy individuals and plays a crucial role in facilitating carbohydrate metabolism. Studies have revealed that a reduction in *Lachnospira* species among pediatric patients with fatty liver disease may exacerbate insulin resistance and inflammatory reactions by reducing the biosynthesis of flavone and flavonol, thereby providing potential therapeutic targets [35]. Recently, there has been a discovery of an increased abundance of *Fusimonas intestini*, belonging to the family *Lachnospiraceae*, within the intestines of both humans and mice exhibiting obesity and elevated blood glucose concentrations. *F intestini* possesses the capacity to synthesize long-chain fatty acids, such as elaidate, under HFD induction. This led to disruption of intestinal barrier integrity, initiation of chronic inflammation, and triggered metabolic endotoxemia, ultimately contributing to the development of obesity and T2DM [36]. Multiple evidences have suggested a significant correlation between *Oscillospira* and leanness, and an association between the reduced abundance of *Oscillospira* and obesity, as well as obesity-related chronic inflammation and metabolic diseases, indicating a genetic predisposition for lower BMI [37,38]. Therefore, *Oscillospira* holds potential as a promising candidate for the next generation of probiotics, exhibiting functions such as weight loss, lipid reduction, and alleviation of MS, and a healthy diet contributed to an increase in the abundance of *Oscillospira* [39]. At the phylum level, numerous studies reported an increased abundance of Firmicutes and a decreased presence of Bacteroidetes in individuals with obesity, both children and adults [9,40]. However, a meta-analysis revealed that individuals with obesity exhibited a reduction in Firmicutes within the gut microbiota, while the concentrations of Bacteroidetes remained unchanged [41]. Therefore, it has been proposed that compared with Bacteroidetes, Firmicutes may serve as a reliable predictor of obesity and play a more pivotal role in facilitating calorie storage and weight gain [42]. The genetic traits of the human gut microbiota demonstrated a higher degree of individual specificity compared with their relative abundance. It has been observed that *Phascolarctobacterium succinatutens* within the Firmicutes achieves an accuracy rate of 88% in identification, and its presence is inversely correlated with body weight and fat mass. Additionally, the study revealed higher concentrations of the genus *Phascolarctobacterium* in individuals with a predisposition to weight loss, suggesting its potential as an indicator for predicting obesity [34]. Craig et al. [43] investigated the correlation between the oral and gut microbiota in 2-y-old children, revealing a decline in the diversity of oral microbial communities and an increase in the ratio of Firmicutes to Bacteroidetes (F/B) with increasing weight. However, establishing a direct correlation between the F/B ratio and specific health conditions remains challenging due to methodologic discrepancies in sample processing and DNA sequence analysis, as well as a lack of consideration for lifestyle factors known to influence microbial composition and diversity. Multiple studies found that overall changes in the F/B ratio between healthy individuals and those with obesity did not exhibit statistically significant differences [44,45]. In general, the gut microbial community of children undergoes dynamic changes. Numerous studies have reported that a significantly higher abundance of Firmicutes, in children with obesity than that in their normal weight counterparts. Firmicutes are known to promote food absorption, fat production, and SCFA production. Additionally, individuals with obesity exhibit lower diversity of microbial communities in the gut than that in normal individuals [[7], [8], [9]]. At the genus level, there was an observed increase in the abundance of anaerobic genera associated with inflammation promotion and complex carbohydrate degradation in the intestinal tract of children with obesity. Conversely, children of normal weight exhibited a significantly higher number of genera related to energy expenditure, modulation of intestinal barrier function, improvement of insulin sensitivity and fat deposition, as well as exerting an anti-inflammatory effect. However, it should be noted that considerable variation exists among individuals regarding the relative abundance of these bacterial genera due to factors such as dietary habits, maternal influence, geographical location, and age.

The gut microbiota composition of children with obesity is intricate, as evidenced by diverse microbial classifications, wherein multiple factors such as race and gender contribute to the development of childhood obesity. Therefore, the identification of specific alterations in the composition or abundance of gut microbiota holds promise for predicting obesity status. Based on this, researchers at the Mayo Clinic in the United States have proposed a new concept known as the gut microbiome health index [46]. This taxonomic map at the species level was constructed by analyzing metagenome from fecal samples, wherein researchers compared the relative abundance of relevant microbial species in 2 cohorts: healthy individuals and those with conditions such as colon cancer, diabetes, cardiovascular disease, or obesity. Ultimately, 50 microbial species that contributed to a healthy microecosystem were identified and categorized into groups representing healthy and unhealthy conditions [46]. Despite significant alterations in fecal microbiota composition due to obesogenic diets, it remains challenging to identify which individuals are susceptible or resistant to obesity. However, analysis of fecal metaproteome has demonstrated variations in protein function and classification associated with susceptibility to obesity, suggesting a potential role for fecal microbial proteome in predicting diet-induced obesity [47]. Therefore, the fecal metaproteome is a potential microbial biomarker for forecasting diet-induced obesity. Furthermore, researchers have conducted a comparative analysis of gut microbiota composition between individuals with obesity and metabolically healthy individuals by using the publicly accessible database of The American Gut Project [48]. They have successfully elucidated the distinctive characteristics of gut microbiota in individuals with obesity and developed machine learning models based on gut microbiota to accurately predict obesity status within populations [[49],^50^]. These findings establish a theoretical framework for intervening in obesity by manipulating the gut microbiota, aiming to gain further insights into the intricate interplay between obesity and gut microbiota. Over the past 2 decades, significant progress has been made in understanding biological behavior, particularly within microbial communities, through the utilization of dynamic models [51]. The utilization of these models enables the exploration of biological complexity at various levels, from gene expression to evolutionary outcomes. Given the dynamic nature of biological systems, there exists a close relationship between dynamical models and predictive biology. However, certain challenges still hinder the full potential impact of predictive biology. Addressing these challenges will undoubtedly require interdisciplinary efforts to advance experimental and computational techniques in microbiology beyond current state-of-the-art levels.

## Factors Influencing the Gut Microbiota and Childhood Obesity

### Early-life factors

**FIGURE 1:**
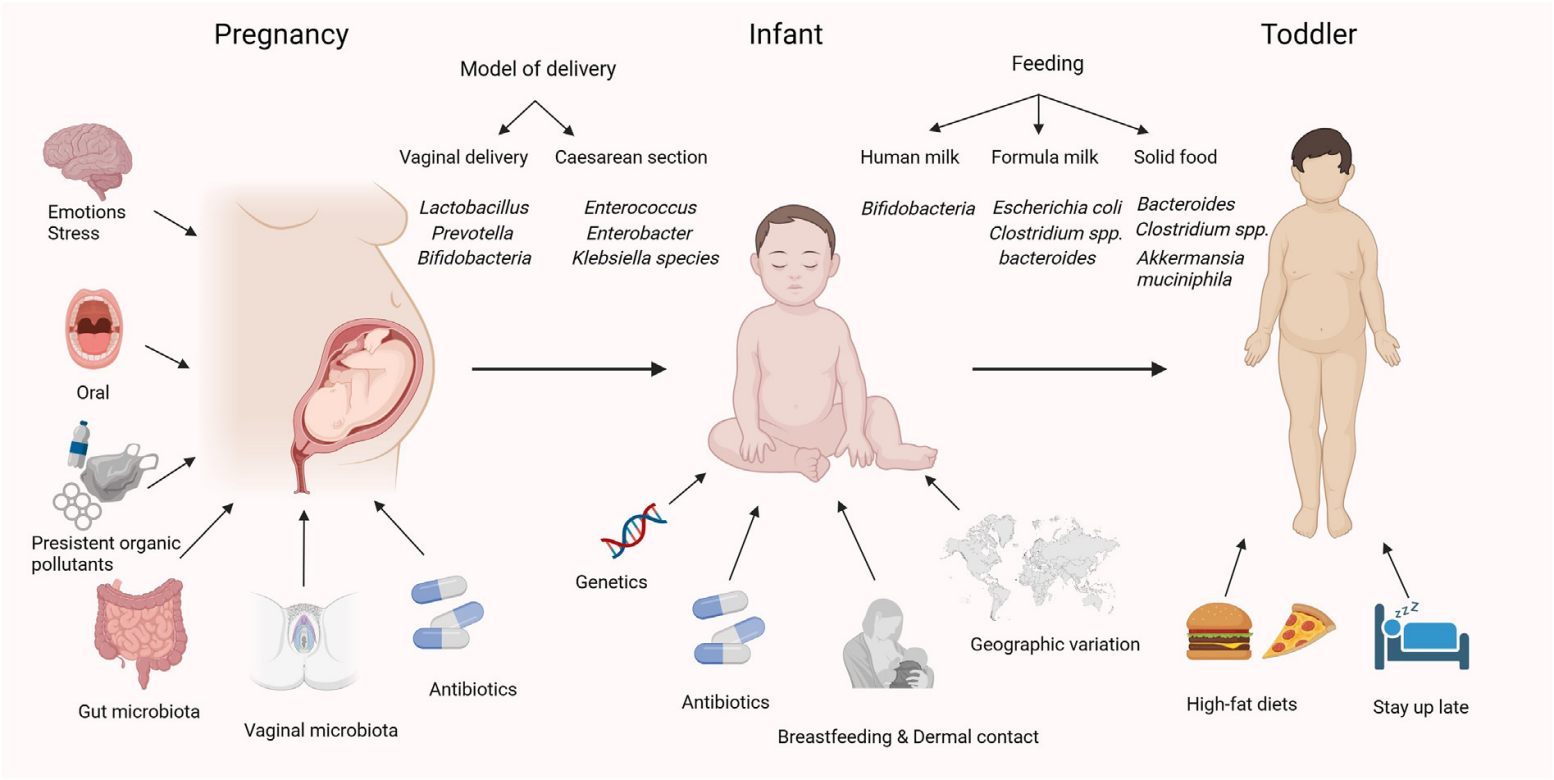
Factors shaping the gut microbiota of infants and young children. During pregnancy, exposure to persistent organic pollutants would impact infant lipid homeostasis in the blood and liver postnatally, and periodontitis or vaginal infections can lead to bacterial invasion into the uterine environment, while emotions can indirectly impact fetal development through the gut–brain axis. The administration of antibiotics during pregnancy and infancy can impede the ability of the dominant microbiota to colonize the skin surface of the newborn. The mode of delivery determines the initial colonization of the infant gut microbiota. Postnatal factors including antibiotic usage, feeding practices (e.g. breastfeeding, formula feeding, and introduction of solid foods), geographic variation, genetics, and environmental exposures further contribute to the configuration of the microbiota during early life. As children age and their diets and environments diversify, the gut microbiota gradually transitions to an adult-like composition, typically by the age of 3.

The colonization of gut microbiota initiates during the fetal period or shortly after birth, persisting until ∼3 to 4 y of age when it reaches stability. The disruption of gut microbiota during this period will have an impact on weight gain and the development of obesity in individuals at the age of 12–14 y [40]. Moreover, the perinatal period and early life span represent critical windows for the establishment of gut microbiota colonization. Among them, full-term vaginal delivery and exclusive breastfeeding, and avoiding exposure to antibiotics, are widely recognized as essential factors for optimal microbial colonization in newborns [52]. The abundance and diversity of gut microbiota in infants and young children are influenced by various factors, including genetic factors, exposure to intrauterine microbiota, adverse prenatal and postnatal events, mode of delivery, feeding, and antibiotic usage, as demonstrated in Figure 1. Of notice, these factors are primarily affected by mother–infant contact, whereas school-aged children are influenced by a wider range of factors, including diverse dietary patterns and living environments [53].

#### Genetics

The occurrence and development of childhood obesity are influenced by various factors, including genetic susceptibility and environmental influences. Recently, it has been suggested that the expression of obesity-associated polymorphic genes in primary human colonic epithelial cells, such as the phosphatidylinositol-4-phosphate 5-kinase (*PIP5K*) type 1A gene, is influenced by exposure to the gut microbiota [54]. The *PIP5K1A* gene, serving as a pivotal regulator in the PI3K/AKT signaling pathway, plays indispensable roles in various biological processes encompassing cellular differentiation, migration, and spermatogenesis. In a rat T2DM model, there was an observed upregulation in circPIP5K1A and α-enolase (ENO1), and downregulation in miR-552-3p, while downregulating circPIP5K1A or upregulating miR-552-3p reduced blood glucose and lipid concentrations and inhibited inflammation. The mechanism lies in the reduction in circPIP5K1A concentrations ameliorates insulin resistance and lipid metabolism disorders, concurrently suppressing inflammation via precise modulation of ENO1 expression facilitated by miR-552-3p [55]. Additionally, a study highlighted a novel involvement of circPIP5K1A in the interplay between miR-552-3p/JAK1/STAT3 pathways within pancreatic β cells, making it a promising therapeutic target for T2DM [56]. Furthermore, the absence of *PIP5K1C* in mouse adipocytes has been found to diminish adipose tissue quality under HFD conditions and ameliorate insulin resistance in vivo, and loss of *PIP5K1C* function impairs adipogenesis in vitro. These findings provided novel insights into the role of *PIP5K1C* in obesity and related metabolic disorders [57]. Genome-wide association studies have revealed a significant association between *A muciniphila* and variants near the phospholipase (PL) D1 gene (*PLD1*) (rs4894707), which exhibited a positive correlation with BMI [58]. Of notice, the involvement of *PLDs* in signal transduction across multiple cellular processes, including cell proliferation, differentiation, cell apoptosis, and immune response, has been elucidated, and the loss of *PLD1* or *PLD2* activity contributes to the development of obesity and T2DM [59]. Furthermore, a significant correlation has been established between the genus *Prevotella* abundance and variation at rs878394 of the human lysophospholipase like 1 gene, which was known to be associated with body fat distribution and insulin sensitivity [60]. Similarly, SCFAs have been demonstrated to elicit epigenetic modifications. Recently, Guo et al. [61] discovered a significant reduction in the total production of SCFAs by the gut microbiota of individuals with obesity, accompanied by an elevated proportion of propionate, which subsequently increased the susceptibility of individuals with obesity to T2DM through its induction of specific DNA methylation at the cg26345888 site. Previous studies have demonstrated a significant correlation between the methylation concentrations of Toll-like receptor (*TLR*) genes and BMI [62]. The activation of *TLR* genes, particularly *TLR4*, plays a pivotal role in obesity by regulating protein phosphorylation and promoting the production of TNF-α, IL-6, leptin, resistin, and chemokines. Among them, TNF-α could facilitate the nuclear factor κB pathway activation, which ultimately hinders glucose transporter protein glucose transporter 4 expression while simultaneously elevating free fatty acid (FFA) concentrations, thereby leading to reduced insulin sensitivity [32]. A study uncovered a notable disparity in the upregulation amplitude of *TLR* genes such as *TLR1*, *TLR4*, *TLR5*, *TLR8*, *TLR9*, and *TL12,* between diet-induced obese mice and leptin-deficient (*ob/ob*) mice. This discrepancy highlighted the potential contribution of enhanced expression levels of TLRs and their related proinflammatory signaling molecules within adipose tissues to the occurrence of metaflammation linked with obesity [63]. Current findings indicated that there existed a disparity in the Bacteroidetes-to-Lactobacillus ratio among individuals with obesity, accompanied by decreased methylation levels of *TLR4* and *TLR2* genes [64]. Analysis of the microbial community of *TLR2* knockout mice demonstrated a decrease in Proteobacteria and Bacteroidetes, an increase in Firmicutes, and elevated concentrations of *Oscillospira* and *Ruminococcus* spp. Furthermore, heightened circulating lipopolysaccharide (LPS) and subclinical chronic inflammation were observed in *TLR2* knockout mice [65]. Additionally, a recent study revealed that fecal micro-RNA (miRNA) can selectively enhance the growth of *Enterococcus* species in a sequence-dependent manner, thereby exerting an influence on the composition of gut microbiome structure. Host-synthesized miRNAs can penetrate bacteria such as *Fusobacterium nucleatum* and *E coli*, thus specifically modulating bacterial gene transcription to impact their proliferation [66]. These findings highlight the role of miRNAs in mediating reciprocal interactions between the host and microbiome.

Certainly, the gut microbiota and their metabolites may serve as essential mediators in the regulation of obesity-related genes. Zhang et al. [67] have revealed that the therapeutic target for Prader-Willi syndrome lies not within human genes but rather within the gut microbiota, which can be accomplished through the augmentation of beneficial bacteria such as bifidobacteria. Researchers at the University Medical Center Groningen in the Netherlands identified 19 heritable bacterial groups and 31 loci that exerted influence on microbial characteristics through genetic sequence analysis conducted on a cohort of nearly 20,000 individuals. Among them, the lactase (*LCT*) gene and fucosyltransferase 2 (*FUT2*) gene significantly contributed to shaping the human gut ecosystem [68]. It has been found that increased lactose intake in individuals with the lactase nonpersistent genotype is correlated with an elevation in the abundance of the lactic acid bacteria and Lachnospriaceae family, as well as alterations in circulating metabolites such as heightened indole propionic acid concentrations and modified branched-chain amino acid metabolism [69]. Analysis of data from the Hispanic Community Health Study/Study of Latinos has revealed reduced risk of T2DM associated specifically with higher milk consumption among individuals possessing the lactase nonpersistent genotype, which is determined by a variant (SNP rs4988235) within the *LCT* gene [70]. The *FUT2* gene can influence the susceptibility to various intestinal disorders, including IBD. In individuals with active α-1, 2-L-fucosyltransferase enzyme, the intestinal mucosa is extensively fucosylated, providing abundant endogenous sources of fucose for symbiotic bacteria. Within the infant gut, specific members of the *Bifidobacterium* genus (such as *Bifidobacterium longum*) metabolize 2'-fucosyllactose derived from breast milk. Infants with lower concentrations of *Bifidobacterium* sp. exhibit reduced fecal oligosaccharide concentrations and elevated lactate concentrations than those with higher concentrations [71]. A comprehensive understanding of the genetic and epigenetic factors influencing host–microbiome interactions is crucial for developing personalized strategies to prevent and manage obesity, as well as its associated comorbidities.

#### Intrauterine microbiota exposure

The sterile womb hypothesis has been challenged in recent years by emerging perspectives, which suggest the potential presence of bacteria in the placenta, amniotic fluid, and fetal membranes [72]. Das et al. [73] discovered that the uterine–fetal transmission of gut microbiota occurs in pregnant women, with the detectable presence of bacteria in the meconium, suggesting the potential existence of intrauterine microbiota. Some scholars have also observed that the composition of the human placental microbiota is similar to that of the oral microbiota and thus hypothesized that the translocation of oral microbiota may contribute to the origin of placental microbiota [74]. However, upon comprehensive analysis of placental samples from 537 women using whole genome sequencing, the researchers observed an absence of microbial colonization in the placenta during a healthy pregnancy [75]. Through the utilization of 16SrRNA gene sequencing technology and shotgun metagenomics sequencing, a comparative analysis was conducted on the microbiota present in 20 term and 20 spontaneous preterm deliveries, no conclusive evidence supporting the existence of a consistent placental microbiota was found in either term or spontaneous preterm placentas [76]. Irrespective of whether the pregnancies were complicated or uncomplicated, the majority of placental samples showed no evidence of bacterial presence. Nearly all signals indicating bacterial detection were attributed to potential contamination originating from bacteria acquired during delivery or laboratory reagents [77]. Overall, existing scientific evidence does not provide support for the existence of a microbial community in a healthy fetal environment. It is noteworthy that placental bacterial infection can still occur under specific circumstances, such as maternal sepsis, which is a prevalent cause of adverse pregnancy outcomes and often results in severe consequences including fetal malformation, miscarriage, and preterm birth. Approximately 15% of neonatal deaths were attributed to neonatal sepsis, with group B streptococcal infection being the primary cause of early-onset sepsis [78].

Additionally, the maternal gut microbiota may impact the metabolic health of offspring. The offspring of conventionally raised female mice gain weight rapidly after exposure to HFD, accompanied by high body fat and hyperlipidemia. In contrast, supplementation of dietary fiber in the maternal diet during pregnancy was less likely to induce obesity even when offspring are HFD fed. This effect may be attributed to the promotion of propionate production by maternal gut microbiota and its subsequent translocation into the embryo through the maternal bloodstream [79]. Although the findings from the animal experiments suggest that supplementation of dietary fiber in pregnant mice may potentially mitigate metabolic disorders, such as obesity, in their offspring, further investigation is warranted to ascertain the generalizability of these conclusions to humans. A study on gestational weight gain revealed that every kilogram of maternal overnutrition-induced gestational weight gain was associated with an increase in the offspring’s BMI, indicating that any form of overnutrition elevates risk of obesity in offspring, irrespective of maternal dietary composition [80]. Undoubtedly, the prolonged administration of antibiotics during the perinatal period not only impedes the colonization of the predominant microbiota on the neonate’s skin surface but also exerts a profound influence on the metabolism of the offspring. According to Mueller et al. [81], the offspring of mothers who received antibiotics during the second or third trimester of pregnancy demonstrated an 84% elevated risk of developing obesity, accompanied by increased BMI and waist circumference.

#### Adverse prenatal and postnatal events

Adverse prenatal and postnatal events, such as exposure to environmental chemicals during gestation, intrauterine growth restriction, and postnatal catch-up growth (CUG), can augment the susceptibility of children and adolescents to metabolic disorders. A study involving 1277 infant subjects used high-throughput 16S rRNA gene sequencing to evaluate their gut microbiota composition. The findings revealed that neonates exposed to environmental smoke exhibited higher relative abundances of *Ruminococcus* and *Akkermansia* species. Exposure to environmental smoke during pregnancy or postpartum increased bacterial richness in infant gut microbiota, especially within the Firmicutes at 3 mo of age, while 6-mo-old infants born to smoking mothers demonstrated elevated abundances of *Bacteroides* and *Staphylococcus* species [82]. Studies have established a connection between an increased abundance of Firmicutes at 3 mo old and elevated risk for overweightness and obesity in children at 1 y old and 3 y old, respectively. The increased prevalence of childhood overweightness and obesity, which was associated with maternal prenatal tobacco smoking, can be attributed to the diversity of Firmicutes resulting in excessive butyrate production [83]. Regrettably, quitting smoking during pregnancy does not mitigate risk of offspring overweightness. Limited existing evidence suggests a necessity for further large-scale longitudinal studies to explore the impact of maternal smoking and environmental tobacco smoke on infant gut microbiota composition as well as its relationship with childhood overweightness.

Maternal exposure to glyphosate had a significant impact on the composition of the gut microbiota in female offspring, leading to a reduction in *Akkermansia* sp. abundance and an increase in the abundance of *Alistipes* and *Blautia* genera. These bacterial alterations were associated with tryptophan metabolism, which was implicated in depression and anxiety disorders [84]. Furthermore, exposure to 2,2',4,4'-tetrabromodiphenyl ether during gestation and lactation can exacerbate obesity, hepatic steatosis, and liver damage in mice induced by HFD. Previous in vitro studies have indicated a potential obesogenic effect of triphenyl phosphate (TPHP) [85]. However, the precise role of TPHP in obesity and associated metabolic disorders remains unclear. In an investigation assessing the impact of prenatal and lactational exposure to 3 doses of TPHP on obesity and metabolic dysfunction in adult male mice fed either a low-fat diet or HFD, the findings demonstrated that TPHP exposure resulted in elevated body weight, liver weight, fat mass, hepatic steatosis, impaired glucose homeostasis, and insulin resistance by modulating mRNA concentrations of genes involved in lipid metabolism with particular emphasis on lipogenesis and lipid accumulation [86]. Moreover, exposure to persistent organic pollutants, such as polybrominated diphenyl ethers and polychlorinated biphenyls, during pregnancy may lead to a long-term and sustained reduction in circulating lipids among children, contributing to pediatric NAFLD through increased lipid uptake [87]. These research findings suggest that prenatal exposure to environmental pollutants has significant impacts on children’s metabolism, potentially influencing the development of public health policies such as environmental monitoring. However, it is crucial to overcome limitations in terms of sample size and potential confounding factors when investigating the long-term effects of environmental pollutant exposure on children’s metabolism, even extending into adulthood.

Currently, dysfunction of the gut microbiota has emerged as a pivotal factor influencing placental insufficiency, a prominent etiology underlying fetal growth restriction (FGR). In patients with FGR, an overgrowth of the genus *Lactobacillus* and the family Erysipelotrichaceae has been observed, with the genus *Catenibacterium* exhibiting a significant negative correlation with fetal growth indicators such as bronchopulmonary dysplasia [88]. Furthermore, recent studies have reported significant alterations in the composition of placental microbiota in FGR newborns, including an increased expression of *Faecalibacterium* and *Lachnospira*, which potentially contribute to the development of FGR, while the family Neisseriaceae may serve as a promising therapeutic target for FGR treatment [89]. Approximately 85%–90% of small for gestational age (SGA) infants exhibited CUG within the first 2 y after birth [90], and global epidemiologic data suggested that CUG in SGA infants may lead to insulin resistance [91]. By inducing maternal food restriction during pregnancy, an SGA rat model was established, and rats were categorized into CUG-SGA and non–CUG-SGA groups based on weight and length at the fourth postnatal week. The results revealed significant differences in gut microbiota diversity and composition between these 2 groups, particularly highlighting the significantly decreased relative abundance of *Lactobacillus* species in the CUG-SGA group [92]. It is imperative to prioritize early monitoring of physical development in children with SGA and also promptly identify signs indicative of accelerated growth during early childhood. This approach can assist in formulating pertinent preventive strategies targeting the mitigation of long-term metabolic risks associated with rapid growth.

#### Mode of delivery

The mode of delivery is one of the key factors influencing the vertical transmission of gut microbiota. To date, the results of the largest genomic study on neonatal microbiota have revealed significant differences in gut microbiota composition between infants delivered vaginally and those born via cesarean section [93]. In infants delivered vaginally, the composition of the neonatal gut microbiota resembled that of the maternal vaginal and skin microbiota, characterized by a predominance of *Lactobacillus* predominating, followed by the genus *Prevotella*. Bifidobacteria becomes dominant during postnatal days 4–7, exhibiting the highest relative abundance. These microbial communities, including *Bifidobacterium* and *Lactobacillus* species, are recognized as key colonizers that play a crucial role in immune programming and the establishment of symbiotic health with the human host in early infancy. *Bifidobacterium* species can synthesize fucosyllactose, acetate, propionate, and 1,2-propanediol through cross-feeding interactions that support the expansion of microbial communities. This facilitates the promotion of immune tolerance toward symbiotic bacteria [94]. Newborns delivered via cesarean section have a colonizing microbiota originating from both maternal skin and hospital environment, characterized by the presence of *Enterococcus*, *Enterobacter*, and *Klebsiella* species [95]. Lee et al. [96] observed a low abundance and diversity of Bacteroidetes and a high diversity of Firmicutes in the intestines of neonates delivered via cesarean section. Previously, a 16-y longitudinal cohort study revealed significantly higher risk of obesity development in offspring delivered via cesarean section than that in those delivered vaginally, whereas mothers with a history of cesarean delivery exhibited 31% lower risk of obesity in offspring delivered vaginally than in those born through consecutive cesarean deliveries [97]. Blustein and Liu [98] reported that neonates delivered via cesarean section exhibited 1.83-fold higher risk of developing obesity at the age of 11 than that of those born vaginally. Additionally, a prospective study discovered that cesarean delivery was associated with significant weight gain in both male and female infants within the Yucatec Maya population, while only boys born via cesarean delivery exhibited notable weight gain in the Toba/Qom region. These findings suggest that although cesarean delivery may contribute to offspring weight gain, it is crucial to consider additional factors such as geographical location and gender [99].

Nevertheless, some scholars proposed that there exists no significant association between cesarean delivery and childhood obesity. Riva et al. [9] discovered that children born via cesarean section weighed more than those born through vaginal delivery. However, the difference was not statistically significant. Controversy persists regarding the association between cesarean section delivery and the subsequent risk of obesity development in adulthood among offspring. In general, multiple retrospective and prospective cohort studies have indicated that children delivered via caesarean section may face an elevated likelihood of developing obesity during childhood compared with those born vaginally [97,[100], [101], [102]]. Similarly, the majority of studies have consistently demonstrated an association between cesarean section and higher risk of adult obesity when compared with vaginal delivery [103,104]. Moreover, meta-analyses and cohort studies have demonstrated a correlation between cesarean section and obesity in young adulthood (18–25 y of age), while controlling for maternal prepregnancy BMI as a confounding factor [105,106]. The primary confounding factor in elucidating the association between mode of delivery and obesity was the maternal prepregnancy weight, aligning with previous studies on the genetic underpinnings of obesity and the impact of maternal obesity on fetal well-being. Consequently, the impact of genetic characteristics in utero and the exposure to obesogenic-related factors may outweigh the influence of the maternal mode of delivery when assessing offspring susceptibility to obesity. Of notice, there has been a statistically significant increase in the prevalence of caesarean sections across all regions since 1990, with East Asia experiencing the most substantial surge. Presently, the global rate of cesarean section stands at 21.1% among women, and projections suggest an estimated rise to 28.5% by 2030 [107]. The etiology of caesarean sections is changing, necessitating the enhancement of relevant measures to effectively reduce the rate of caesarean sections. This includes bolstering midwifery techniques and ensuring safe delivery management, with a particular emphasis on allocating resources for vaginal deliveries, and simultaneously strengthening public awareness and educational campaigns.

In summary, the colonization of the infant gut microbiota at birth is a critical process, and the initial months of life play a pivotal role in the establishment of transgenic elements and immune system maturation. Disruption of transgenic components can contribute to various metabolic and allergic disorders, such as obesity, diabetes, and Crohn’s disease. Recent research has demonstrated that delivery mode significantly impacts the presence of transgenic components in neonates [108]. The administration of probiotics and prebiotics to infants delivered via cesarean section can partially restore alterations in fecal microbiota composition, indicating that the mode of delivery may act as a proxy or mediator covariate rather than an independent determining factor in establishing postpartum microbiota [109]. It is evident that the mode of delivery plays a significant role in shaping postpartum maternal gut microbiota; however, it may only tell part of the story. Conducting a more comprehensive investigation into all prenatal and perinatal factors that could potentially influence neonatal microbiota will contribute to a better understanding of diverse risks and pathophysiology associated with common chronic childhood diseases, as well as adverse adult health outcomes.

#### Feeding

The gut microbiota of breastfed infants is dominated by bifidobacteria, which serve as markers of a healthy infant gut microbiota and have demonstrated beneficial effects on glucose tolerance and reduction of intestinal inflammation. In contrast, in formula-fed infants, the dominance of bifidobacteria as the microbial community in the intestinal tract was diminished, concomitant with an elevated proportion of bacteria such as *E coli* and *Clostridium spp* [110]. While formula cannot fully replace breastmilk, optimizing the utilization of formulas fortified with essential components resembling those found in breastmilk would confer greater advantages to infant health. Human milk oligosaccharides, as one of the predominant constituents in breast milk, selectively modulated beneficial bacteria by promoting the proliferation of *Bifidobacterium* and *Lactobacillus* spp in infants’ gastrointestinal tract [111]. However, for certain mothers, formula feeding may be the only viable alternative. In such cases, formula milk fortified with oligosaccharides was considered as the optimal substitute. A randomized controlled trial conducted by Liber et al. [112] demonstrated that a combination of 3 oligosaccharides could augment bifidobacteria abundance in infant gut microbiota compared with solitary prebiotic usage. For mixed-fed infants, their gut microbiota exhibited a stronger resemblance to those exclusively fed with formula milk. The introduction of formula alongside breastfeeding might contribute to an elevated prevalence of Firmicutes and underlies heightened risk of early-onset obesity associated with feeding patterns [113]. Before the introduction of solid food, infants possess the inherent ability to digest plant polysaccharides. Laursen et al. [114] noticed that with weaning and the addition of solid complementary foods, infants gradually acquired a gut microbiota resembling that of adults, characterized by colonization of *Clostridium* spp. and *A muciniphila*, which progressively increases with age.

#### Antibiotics usage

In recent years, the impact of low-dose antibiotics on the health of pregnant women and fetuses has emerged as a prominent research issue. Of particular concern is the presence of residual antibiotics from animal sources in food. Commonly detected residual antibiotics in animal-based foods include β-lactams, tetracyclines, aminoglycosides, and macrolides. Studies have revealed that continuous exposure to low-dose antibiotics during pregnancy can induce alterations in the α-diversity of neonatal gut microbiota and significantly diminish the abundance of predominant genera such as *Bacteroides* and *Ruminococcus*, while promoting dominance of *Streptococcus* [115,116]. Furthermore, the transmission of low-dose antibiotics from the maternal to newborns via breast milk can impact their gut microbiota composition [117]. Exposure to these antibiotics disrupted the composition of the mouse gastrointestinal microbiota and impaired its metabolic capacity, particularly by elevating concentrations of SCFAs that directly fuel colon cells and were absorbed into the portal vein circulation. This stimulation ultimately promoted adipogenesis in mice, gradually leading to obesity [118]. Currently, animal studies have demonstrated that administration of low-dose penicillin during late pregnancy and early postnatal period exerted significant effects on the offspring of mice. Specifically, it has been observed that penicillin induces persistent alterations in the gut microbiota composition (elevated concentrations of *Murinus Lactobacillus* and *Klebsiella* species), upregulated cytokine expression in the frontal cortex, and disrupted the integrity of the blood–brain barrier. Furthermore, mice treated with antibiotics exhibited anxiety-like behavior and impaired social interactions [119]. The persistent exposure of pregnant women to low-dose antibiotics exerted a cumulative impact on the gut microbiota of newborns, resulting in enduring consequences. Even after discontinuing low-dose penicillin treatment for several weeks, the gut microbiota in mice from the experimental group exhibited recovery. However, these mice still developed obesity as adults, which indicates irreversible consequences [120]. These findings highlight the detrimental impact of prenatal exposure to low-dose antibiotics on various physiologic systems, including neurology and metabolism, as well as subsequent growth and development in offspring. Further investigation is warranted since current studies exploring the influence of low-dose antibiotic exposure during pregnancy on neonatal gut microbiota primarily rely on animal models.

The period from birth to 6 mo is when the gut microbiota is more sensitive to antibiotic exposure. A multinational team of European researchers has revealed that early-life administration of antibiotics is associated with an increased susceptibility to childhood obesity, particularly among male individuals [121]. Gu et al. [122] evaluated the short-term effects of fluoroquinolone and β-lactam antibiotics on the gut microbiota of mice by 16S rRNA gene sequencing and showed that only 4 d of antibiotic exposure significantly diminished both α and β diversity of gut microbiota. There is still controversy about the correlation between early antibiotic use and childhood obesity. Some scholars believe that early antibiotic exposure may have a cumulative effect on the development of obesity, contingent upon the frequency and duration of antibiotics. Conversely, a randomized controlled trial involving 607 children found no significant correlation between early-life antibiotic exposure and the occurrence of overweight or obesity in children. The researchers concluded that retrospective studies only assessed the exposure effect of incidental antibiotic use, and available evidence remained confined to observational studies with limited levels of evidence [123]. Overall, growth and development in children is a continuous and irreversible process, and the response and recovery of gut microbiota to antibiotics can be modulated by multiple factors such as the host’s genetic predisposition, immune system functionality, dietary patterns, and drug metabolic processes. Attention should be directed toward the potential impact of antibiotic exposure during critical developmental periods on obesity, with a focus on investigating the correlation and underlying mechanisms between alterations in gut microbiota due to antibiotics and childhood obesity. This could provide a theoretical basis for more prudent use of antibiotics in early life.

### Diet and behavior

Compared with genetic factors, alterations in dietary structure exert a more pronounced regulatory influence on the composition of gut microbiota, predisposing to the colonization of gut microbiota associated with dietary preferences, which represent one of the foremost determinants shaping the microbial community structure [124,125]. Among them, HFD is the main cause of overnourishment in children. Nakayama et al. [126] demonstrated that the Western diet characterized by a high lipid and glucose content, led to a reduction in the genus *Prevotella* abundance within the gut microbiota and contributed to childhood obesity. Conversely, children following a carbohydrate-based diet exhibited an increase in *Prevotella* concentrations and a decrease in *Bacteroides* populations within their gut microbiota. Compared with nonvegetarians, vegetarians exhibited a higher abundance of *Prevotella* and elevated *Prevotella*/*Bacteroides* ratios, which were associated with enhanced glucose metabolism induced by dietary fiber. Studies have shown that high-fat or high-carbohydrate diets can stimulate microglia cells to release IL-1β, IL-6, and TNF-α via the GBA, thereby triggering an inflammatory response in the basal hypothalamus region and leading to leptin resistance within the central nervous system (CNS). Ultimately, this cascade promoted the development of insulin resistance and T2DM. On the contrary, it also activated protease-activated receptors in intestinal epithelial cells, leading to downregulation of the expression of tight junction proteins occludin and zonula occludens-1 within the intestinal epithelium, which compromises the mechanical barrier of the intestinal mucosa and increases the permeability of the intestinal wall, thus causing intestinal inflammatory diseases [127,128]. By observing food intake in adolescent mice (4 wk old) and recording trends in neuronal activity of the brainstem dorsal vagal complex within 24 h, the researchers have discovered that a HFD disrupts the circadian rhythm in the brain responsible for regulating satiety. This disruption was evidenced by a decrease in neural stem cell rhythmicity and an increase in food intake, ultimately leading to the development of overeating and obesity [129]. Currently, certain food additives such as preservatives, emulsifiers, and artificial sweeteners have been revealed to increase risk of childhood obesity by altering the diversity of gut microbiota. Among them, propionic acid and its sodium and calcium salts (organic acid food preservatives) have been identified as a class of metabolic disruptors that can exacerbate risk of T2DM and obesity in humans [130]. Another study demonstrated a significant association between the consumption of artificial sweeteners, including saccharin, sucralose, and aspartame, and impaired glucose tolerance in both mice and humans [131]. Furthermore, emulsifiers such as polysorbate-80 and carboxymethyl cellulose have been found to potentially contribute to mild inflammation, obesity, and other chronic inflammatory diseases [132]. Since children’s body size and dietary intake are different from those of adults, they are more susceptible to the effects of food additives. Therefore, it is advisable for children to minimize their consumption of food additives and actively explore improved product of additives.

The composition of gut microbiota in children under the age of 3 is also associated with their behavioral characteristics. Bacteria present on the surface of the breast skin can directly enter the infant’s gastrointestinal tract during breastfeeding. Pannaraj et al. [133] collected 3 types of samples (breast milk, skin of the areola, and infant feces) from 107 healthy mother–infant pairs and found that breast milk contributed to 27.7% of gut microbiota in infants who consumed >75% of their daily milk intake came from breast milk, while an additional 10.3% came from the skin of the areola. In addition, the behaviors of infants and young children, such as digit-sucking, locomotor development (crawling and walking), manual exploration of the environment, expose them to a higher bacterial load than adults, thereby increasing risk of infection. In modern society, unhealthy lifestyles like sedentary behavior and staying up late also contribute to the promotion of obesity. Hence, implementing effective measures such as reducing the consumption of HFD and sugary beverages, as well as strengthening physical exercise, can effectively prevent childhood obesity.

### Geographic factors

The Guangdong Gut Microbiome Project conducted in 2018 used data mining techniques to reveal that the geographical location of hosts exerted a significantly greater impact on gut microbiota than other factors such as age, disease, and lifestyle. This finding underscored the substantial impact of regional disparities on the variability of gut microbiota within populations [134]. The influence of different geographic locations on variations in gut microbiota composition may originate from disparities in lifestyle and dietary culture. According to reports, a geographic gradient existed in the gut microbiota of European infants, with those from Northern Europe (Sweden, Scotland, and so on) exhibiting higher concentrations of bifidobacteria and *Clostridium* spp., while *Lactobacillus* and *Bacteroides* species were more prevalent among infants from Southern Europe (Spain, Italy, and so on) [135]. Compared with Italian children, the gut microbiota of 1- to 6-y-olds in rural Burkina Faso exhibited a significant reduction in *Firmicutes* and an enrichment in the genus *Prevotella* and xylan-using bacteria. The genus *Prevotella* is commonly observed in individuals who adhere to a vegetarian diet, primarily due to its ability to metabolize noncellulosic polysaccharide and pectinolytic properties. Being the second most abundant indigestible carbohydrate in nature, xylan is used by bacteria to facilitate host digestion. This observation can be hypothesized as a consequence of the African children’s consumption of polysaccharide-rich diets, which shape their gut microbiota to optimize energy intake from plant fibers while simultaneously protecting against intestinal inflammatory diseases and noninfectious inflammation of the colon [136]. In developed countries, like the United States, the abundance of *Firmicutes* and *Actinobacteria* species detected in infant feces is comparatively lower than that observed in African infants [137]. Compared with developing countries, developed countries have superior healthcare and service facilities, which can provide a better environment for the colonization of beneficial bacteria in newborns. Through prolonged coevolution with the host, the gut microbiota has developed a range of protective mechanisms that contribute to host health. However, the phenotypic effects of the microbial community can be context-dependent, exhibiting benefits in certain environments or individuals while being detrimental in others. For instance, *Prevotella copri*, an abundant fiber-degrading agent in nonindustrialized human gut microbiota, has been shown to improve glucose tolerance while potentially exacerbating chronic inflammation depending on the specific environment [138]. The composition of gut microbiota was primarily shaped by environmental factors and cohabitation, only ∼6.6% of taxa were heritable while ∼48.6% can be attributed to cohabitation [139]. The gut microbiota is considered a crucial environmental factor and selective agent influencing mammalian diet, phenotypic plasticity, gastrointestinal morphology, and immune adaptability throughout evolution. Thus, variations in gut microbiota may significantly impact evolutionary outcomes across different interactions between mammalian species and their respective gut microbial communities.

## The Mechanisms Underlying the Impact of Gut Microbiota on Childhood Obesity

### Influence on energy absorption

The gut microbiota and its metabolites play distinct and intricate roles in regulating glycolipid metabolism as well as maintaining the balance between energy supply and demand. Under normal conditions, *Bacteroides* species can decompose complex carbohydrates and plant fibers, thereby facilitating the absorption of monosaccharides and nutrients by the body. An in vivo study conducted in mice via oral gavage has demonstrated that *Bacteroides thetaiotaomicron* can decrease glutamate concentration in mouse serum while enhancing lipolysis and fatty acid oxidation in adipocytes, thereby mitigating fat accumulation [140]. However, a previous animal study observed that when germ-free mice were inoculated with *B thetaiotaomicron* extracted from the intestines of healthy mice for 2 wk, there was a significant increase in body fat by 57%. This increase can be attributed to the ability of *B thetaiotaomicron* to enhance the expression of genes involved in the degradation and absorption of HFD in these mice, ultimately leading to excessive fat absorption within the gut [141]. *B thetaiotaomicron* plays a dual role by simultaneously regulating probiotic mechanisms and pathogenicity, and like other gut microbiota, it maintains a complex and delicate symbiotic relationship with humans. Therefore, it is crucial to adopt a dialectical approach when considering the role of gut microbiota in children.

Normally, glycoside hydrolases responsible for the digestion of plant polysaccharides are limited in humans. However, anaerobic bacteria such as *Lactobacillus* and *Ruminococcus* species can synthesize abundant quantities of these enzymes to degrade dietary fibers and produce SCFAs, including butyrate, acetate, and propionate, in the proximal colon. Of note, Bacteroidetes predominantly produces acetate and propionate, while Firmicutes primarily synthesizes butyrate [142]. The benefits and limitations of SCFAs on human health are primarily determined by the concentration of metabolites or their location within organs. Butyrate serves as the primary energy source for colonocytes, whereas other SCFAs are transported to the liver via the portal vein, acetate can contribute to fatty acid and cholesterol synthesis, and propionate becomes a substrate for gluconeogenesis [143,144]. It is now generally accepted that butyrate is a health-promoting ingredient because of its ability to enhance insulin sensitivity, exert anti-inflammatory effects, regulate energy metabolism, and increase leptin gene expression. Conversely, propionate diminishes the brain’s satiety response to high-calorie foods and necessitates a higher food intake for the same level of satisfaction, thereby promoting overeating and subsequently triggering obesity [145].

SCFAs have been shown to stimulate the proliferation of epithelial cells in both the small intestine and colonic intestines and promote intestinal absorption of nutrients, as well as to regulate peptide tyrosine-tyrosine (PYY) and glucagon-like peptide (GLP)-1 through activation of FFA receptor 2, establishing a negative feedback pathway for energy regulation and appetite suppression [146]. Moreover, SCFAs can also inhibit the secretion of fasting-induced adipose factor, thereby enhancing lipase activity and facilitating triglyceride accumulation in adipose tissue, thus promoting obesity [147]. On the contrary, SCFAs exert their effects on G-protein coupled receptor (GPR) 41 andGPR43, resulting in the inhibition of hepatic lipid accumulation, acceleration of lipid and glucose catabolism in extrahepatic tissues, and improvement of insulin sensitivity [148]. It has also been reported recently that rapid infusion of acetate or a mixture of SCFAs (containing acetate, propionate, and butyrate) into the distal colon of individuals with overweight or obesity resulted in an increased rate of lipid oxidation during fasting and enhanced energy expenditure at rest [61]. By supplementing soluble dietary fiber to increase the concentration of SCFAs in the cecum, thereby augments plasma concentrations of PYY and GLP-1, enhances satiety signaling in the brain, reduces food intake, and ultimately mitigates obesity [149]. In conclusion, the production of SCFAs is significantly influenced by the composition of gut microbiota, and any interference with this delicate balance could lead to an imbalanced production of SCFA species, thereby accelerating the onset of obesity and MS.

### Alteration of metabolic pathways

Metabolites produced by the gut microbiota enter the systemic circulation mainly through intestinal absorption, the enterohepatic circulation, or alterations in intestinal permeability induced by the gut microbiota. As a prominent metabolite derived from the gut microbiota, SCFAs significantly impact adipose tissue metabolism. Acetate, for instance, exerts an inhibitory effect on lipolysis stimulated by beta-adrenergic receptors in adipocytes, and this antilipolytic effect may be attributed to its ability to inhibit the phosphorylation of hormone-sensitive lipases that are dependent on G protein-coupled receptor signaling [150]. Animal models have demonstrated that exogenous supplementation with a mixture of SCFAs inhibits cholesterol synthesis and reduces hepatic fat accumulation in animals, and the underlying mechanisms are associated with an increase in hepatic fatty acids oxidation and a decrease in fatty acid synthase activity, which is mediated by the AMP-activated protein kinase acetyl-CoA carboxylase signaling pathway [151]. Furthermore, accompanied by dysfunction in the gut microbiota, the increased production of metabolites associated with the gut microbiota, such as LPS and succinate, also contributes to metabolic disorders and fosters the development of obesity. LPS, a typical Gram-negative bacterial outer membrane-associated glycolipid, not only induces an inflammatory response but also plays a crucial role in promoting the pathogenesis of diseases such as T2DM and NAFLD by mediating insulin resistance [152]. More, succinate is an intermediate product of propionate fermentation, the proliferation of propionate-producing bacteria (Prevotellaceae and Veillonellaceae), along with the reduction in succinate-consuming bacteria (*Pseudomonas aeruginosa*), leads to an elevated succinate concentration within the body, thereby impeding lipidolysis and promoting lipid accumulation [153].

Bile acids are among the determinants of gut microbiota abundance, diversity and metabolic activity. The gut microbiota possesses the ability to metabolize bile acids, thereby exerting an influence on both lipid metabolism and gut health. During the neonatal period, elevated concentrations of primary bile acids lead to an enrichment of bacteria associated with the expression of genes involved in bile acid metabolism within the neonatal intestine. Moreover, primary bile acids could be metabolized by gut microorganisms to generate secondary bile acids with more intricate roles, including participating in enterohepatic circulation, facilitating the emulsification of dietary fat, and promoting lipid digestion [154]. It has recently been revealed that bile acids possess additional endocrine functions and can serve as crucial signaling molecules [155]. The synthesis of bile acids is negatively regulated by the farnesoid x receptor (FXR), which acts as a feedback mechanism to inhibit bacterial overgrowth and mucosal injury in the ileum by enhancing the expression of genes involved in intestinal protection. Fu et al. [156] discovered that an increase in total bile acid concentrations was accompanied by elevated intestinal permeability in mice fed a HFD, which correlated with a decrease in FXR-mediated zonula occludens 1 protein expression and alterations in cecum and plasma bile acid concentrations. After knockout of intestinal hypoxia-inducing factor 2α, a reduction in the abundance of *Bacteroides vulgatus* and an increase in the abundance of *Ruminococcus torques* were observed in mice, resulting in a significant elevation in the concentrations of taurine binding bile acid and deoxycholic acid. This perturbation triggers the activation of takeda-G protein-receptor (TGR)5 in white adipose tissue, leading to an upregulation in the expression of G protein–coupled receptor-kinase interacting protein 1 and creatine kinase 2 in mitochondria. As a result, thermogenesis is promoted and fat utilization is enhanced, ultimately ameliorating obesity. Moreover, as a crucial receptor involved in the regulation of bile acid motility, TGR5 can be readily activated by microbiota-produced bile acids such as deoxycholic acid and lithocholic acid, which directly act on neurons and indirectly stimulate the release of 5-hydroxytryptamine (5-HT) [157]. Considering the favorable effects of bile acid receptors FXR and TGR5 on obesity, exploration of FXR and TGR5 agonists holds promise as a novel avenue for research in obesity treatment.

Apart from metabolites, the reduction of *A muciniphila* has also been demonstrated to be closely related to the development of T2DM in obese mice, manifested as a decrease in the abundance of *A muciniphila* in the intestines of T2DM mice model. However, when their diets were supplemented with *A muciniphila* or its outer membrane protein Amuc_1100, it prevented the progression of MS induced by HFD and effectively improved the glucose tolerance in mice [158]. It is important to note that excessive supplementation of *A muciniphila* may cause excessive degradation of the intestinal mucosa, thereby increasing risk of leaky gut and allergic diseases.

### Induction of an inflammatory response

The presence of mild systemic inflammation, which is a result of the body’s immune response to LPS, is one of the defining characteristics associated with obesity. Compared with children of normal weight, children with obesity demonstrate an increased relative abundance of proinflammatory gut microbiota (e.g. Verrucomicrobia of Gram-negative bacteria) and elevated concentrations of proinflammatory cytokines within the gut microbiota. This is attributed to the translocation of LPS from Gram-negative bacteria into the liver and adipose tissue via systemic circulation, thereby inducing a chronic low-grade inflammatory response within the body [159,160]. A series of studies have demonstrated that dysfunction of gut microbiota enhances the absorption of LPS, triggers an inflammatory response, and promotes phosphorylation of insulin receptor substrate 1 through insulin signaling pathways such as nuclear factor κB, thereby contributing to the development of insulin resistance and obesity [161,162]. Simultaneously, the endocannabinoid system activity will be upregulated, resulting in the inhibition of appetite suppressors like cholecystokinin, thereby reducing the host’s sensation of satiety and ultimately leading to overeating and obesity [163]. Of note, not all types of LPS have adverse effects on host metabolism. Comparing the LPS dose from *Rhodopseudomonas sphaeroides* and *E coli* using endotoxin units reveals that only *E coli* LPS induces dysglycemia and adipose inflammation, delays intestinal glucose absorption, and enhances insulin and GLP-1 secretion. Metabolically advantageous endotoxemia induced by *R sphaeroides* LPS mitigates dysglycemia provoked by an equivalent dose of *E coli* LPS and improves glucose control in obese mice. In the context of metabolic endotoxemia, the influence of different types of bacterial LPS on intestinal permeability, intestinal glucose absorption, blood glucose concentrations, insulin secretion, and gut incretin response can lead to either favorable or detrimental outcomes [164]. Consequently, when considering interventions such as prebiotics, probiotics, and postbiotics in the management of metabolic endotoxemia, their potential to modulate LPS characteristics should be taken into account.

In addition, a HFD is a nonnegligible contributor to the process of intestinal inflammation induced by altered gut microbiota. By feeding HFD to nucleotide-binding oligomerization domain-containing protein (NOD) 1 and 2 gene knockout mice, Schertzer et al. [165] observed that HFD-induced dysfunction of gut microbiota activated the NODs, thereby promoting NOD-like receptor–mediated regulation of inflammasome formation. This process further accelerated the development of insulin resistance in obese mice through macrophage-derived release of the inflammatory factor TNF-α, although it may also be implicated in innate immunity [165]. However, a randomized controlled trial conducted in Australia on individuals with obesity revealed that the Mediterranean diet led to an increased abundance of *Rothia* and *Treponema* spp, while a complex carbohydrate-rich diet resulted in an increased abundance of *Prevotella* and *F prausnitzii*, both of which enhanced insulin sensitivity [166]. Among them, *F prausnitzii* is regarded as a promising therapeutic candidate owing to its capacity for producing metabolites with anti-inflammatory properties [32]. By supplementing rats on HFD with probiotic preparations (*Bifidobacterium* sp., *Lactobacillus* sp., and *Lactobacillus reuteri*), it was observed that plasma concentrations of LPS and IL-1β were reduced, inflammatory indices decreased, and resulted in improved insulin sensitivity and alleviation of obesity [167]. In conclusion, the supplementation of probiotics and prebiotics may serve as an effective intervention strategy to improve the inflammatory state and promote intestinal health in children.

### The gut–brain axis

**FIGURE 2:**
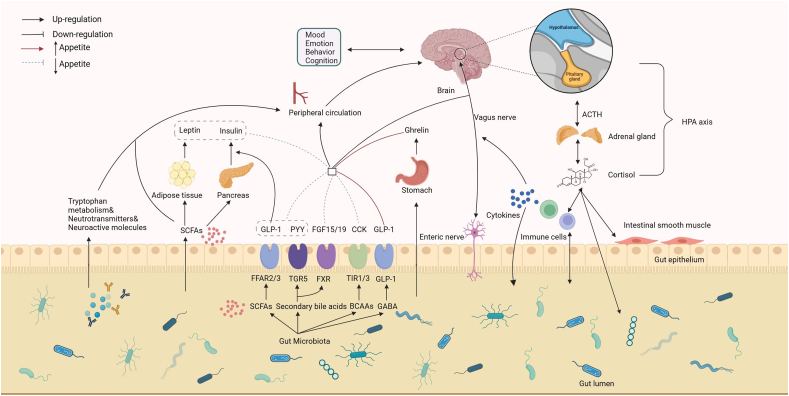
The mechanisms underlying bidirectional communication between the gut microbiota and the brain. The central nervous system is involved in various signaling processes, such as immunomodulatory responses, neuronal innervation, enteroendocrine regulation, and microbial metabolite modulation, through vagal efferent activity and the modulation of the hypothalamic–pituitary–adrenal axis in response to a variety of external stimuli. The gut microbiota is extensively involved in the synthesis and release of various hormones, active metabolites, and active compounds such as neurotransmitters, SCFAs, secondary bile acids, branched-chain amino acids (BCAAs), and γ-aminobutyric acid (GABA), which in turn influence the brain function and host behavior (e.g. regulation of appetite and food intake). On the contrary, the gut microbiota can directly impact gastrointestinal, adipose, and pancreatic tissues to produce appetite-regulating peptides such as leptin, insulin, and gastrin. These peptides reach the brain’s appetite-regulating centers through the peripheral circulation or the vagus nerve and play a crucial role in regulating appetite and food intake. The bidirectional flow of information from the gut–brain axis also modulates the composition of gut microbiota and regulates behavior, mood, and cognition.

The GBA is a sophisticated neurohumoral communication system that is bidirectionally regulated by the gastrointestinal tract and the brain, encompassing the CNS, autonomic nervous system, and enteric nervous system, as well as the hypothalamic–pituitary–adrenal (HPA) axis. The GBA plays a crucial role in regulating immunity, inflammation, and stress response, and it also serves as an important signaling axis for maintaining metabolic balance in humans, as shown in Figure 2. A variety of neuroactive factors produced by gut microbiota have been demonstrated to play a crucial role in mood regulation [168]. In 2017, it was first demonstrated that alterations in gut microbiota can influence mood and social behavior. By collecting magnetic resonance images of the brains from 2 cohorts of female subjects, researchers observed that females with an abundance of the genus *Prevotella* in the intestinal tract exhibited reduced hippocampal activity and were more prone to negative emotions such as anxiety, grief and irritability compared with those with a predominance of *Bacaeroides* in the intestinal tract [169]. In 2021, Reissland et al. [170] discovered a correlation between psychological disorders such as depression and anxiety in pregnant women and the occurrence of low birth weight in newborns. Recent studies on host–microbe interactions in insulin resistance have suggested that this correlation is primarily attributed to the involvement of gut microbiota, especially *Lachnospira* sp., which mediates the reduced secretion of various hormones and neuromediators including insulin-like growth factors [171]. These findings will provide a fundamental basis for further investigating the mechanisms by which the gut microbiota affects the function of the nervous system.

This study suggests that SCFAs can directly modulate the sympathetic nervous system through the activation of FFA receptors, which are involved in regulating blood glucose stabilization and hunger-satiety balance. SCFAs produced by microbial fermentations in the intestinal tract of pregnant women also potentially impact fetal intestinal, pancreatic, and neural development through GPR43 and GPR41 signaling pathways. Within the CNS, SCFAs could facilitate microglia maturation and contribute to their differentiation into other glial cells types [76]. Perry et al. discovered that acetate play a pivotal role in the pathogenesis of obesity in murine models. Following extensive microbial fermentation, the substantial production of acetate is absorbed into the bloodstream, subsequently crossing the blood–brain barrier and activating the parasympathetic nervous system. This activation prompts insulin secretion and initiates the energy storage program. Concurrently, stimulation of the parasympathetic nervous system induces gastric release of ghrelin, thereby eliciting feelings of hunger and augmenting food intake [172]. However, the gut microbiota is also able to regulate the secretion of various appetite-suppressing hormones by intestinal endocrine cells, such as cholecystokinin, GLP-1, and PYY. These hormones transmit signals to the CNS through the neurohumoral circuit, which activates key metabolic sites in the hypothalamus, inducing satiety and reducing food intake [173]. Recent studies have revealed a strong correlation between BCAA metabolism and obesity, and the synthesis of branched-chain amino acids (BCAAs), including isoleucine, leucine, and valine, relies on gut microbiota such as the genus *Prevotella*, *Streptococcus* spp., or their direct intake from dietary sources. Imbalances in the concentrations or ratios of BCAAs can lead to appetite suppression, while a proportionate balance of BCAAs promotes appetite [174]. Canfora et al. [151] discovered that intraperitoneal injection of a combination of SCFAs in murine models exerted an inhibitory effect on energy intake through vagal stimulation of afferents. In all, energy intake and consumption undergo dynamic changes, and any disruption in this equilibrium can result in obesity.

The production of a range of neurotransmitters and neuroactive molecules by the gut microbiota is also essential for the regulation of intestinal function, such as 5-HT, γ-aminobutyric acid, acetylcholine and catecholamines. These molecules possess the potential to impact brain development, and myelin formation, as well as social, emotional, and anxiety-like behaviors [175]. Gut-derived 5-HT is an important component of the GBA, which is essential for mood regulation, hunger, sleep, peristalsis of colon, and secretory activity in the intestinal tract [176]. In addition, as the only precursor of the neurotransmitter 5-HT, metabolites derived from tryptophan in the kynurenine pathway can also regulate neural activity and exhibit activity within the inflammatory cascade [177]. It has been shown that gut microbiota induces the synthesis of 5-HT through SCFAs, thereby influencing the host’s emotional response to food consumption and increasing their inclination toward dietary choices associated with the gut microbiota, promoting the development of unhealthy eating habits and exacerbating disturbances within the gut microbiota [178]. Bhattarai et al. [176] demonstrated that dysbiosis of gut microbiota led to a downregulation of 5-HT receptor gene expression, resulting in the inhibition of 5-HT secretion. This dysregulation further attenuated the regulatory effect of 5-HT on the host’s tryptophan metabolic pathway, ultimately leading to insulin resistance. It has also been revealed that the typical inhibitory neurotransmitter γ-aminobutyric acid in the CNS can stimulate feeding, promote energy accumulation, and trigger the onset of obesity. Moreover, chronic or acute stress states would activate hypothalamic–pituitary–adrenal axis, leading to an increase in intestinal permeability, and facilitating the occurrence of intestinal inflammation. Conversely, the cholinergic anti-inflammatory pathway acting on the vague nerve is beneficial to attenuating the mild systemic inflammation induced by LPS [179]. When there is dysfunction in the vagal signaling of GBA, both metabolites and inflammatory factors can contribute to the development of intestinal disorders, such as IBD and irritable bowel syndrome, through the bidirectional regulation of GBA. Consequently, the concept of the GBA has been established, and there is already evidence that obesity, T2DM, chronic inflammation, and other related metabolic disorders are partly caused by imbalances in the interaction between the host and the gut microbiota or metabolites. However, the precise pathways and mechanistic connections linking the brain, the gut microbiota, and the peripheral target organs remain incompletely elucidated. Therefore, expediting the mapping of gut microbiome genes is imperative to gain further insights into the role of GBA in childhood obesity.

## Treatment of Childhood Obesity From the Perspective of Gut Microbiota

**FIGURE 3:**
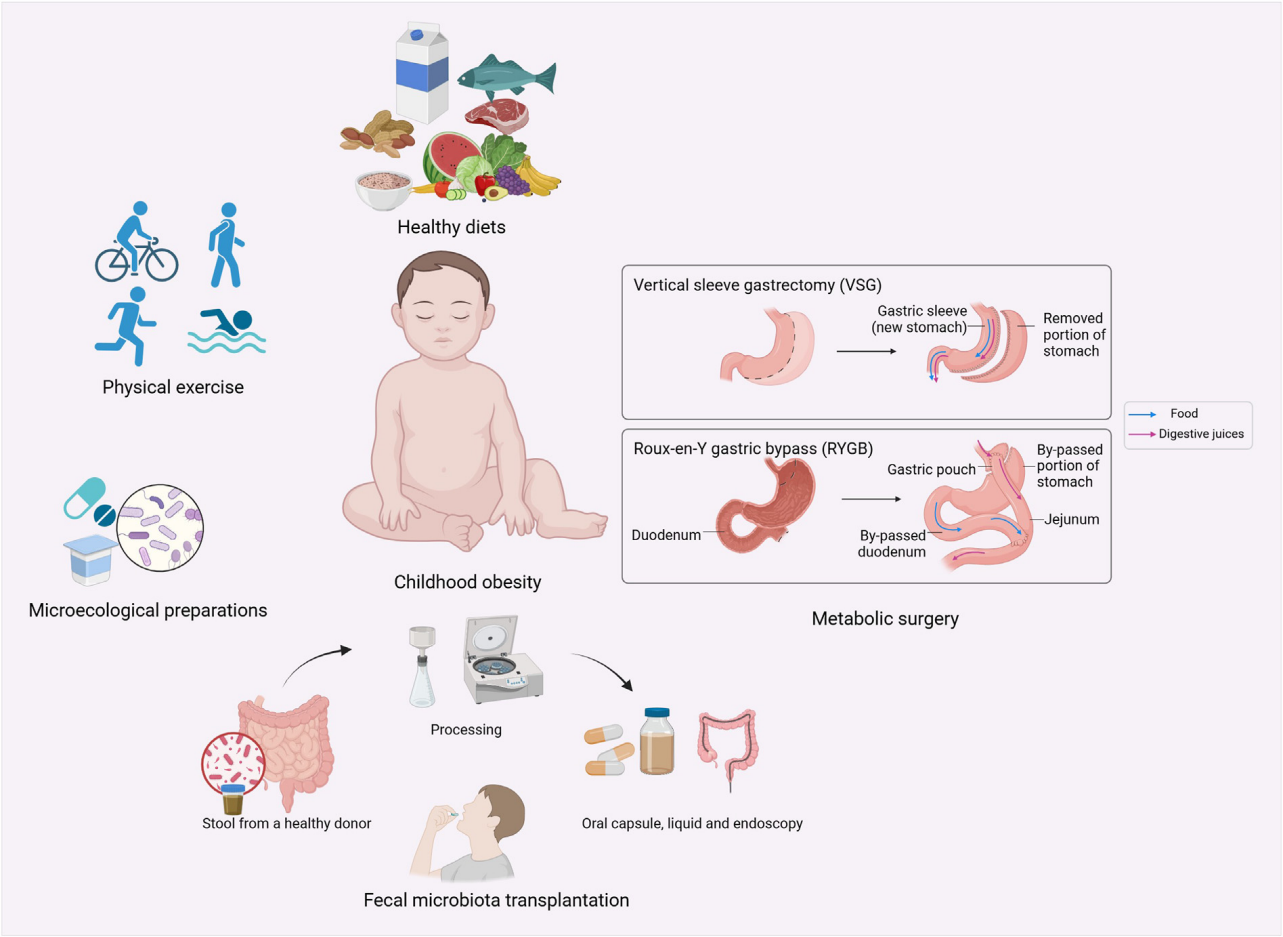
Treatment of childhood obesity from the perspective of gut microbiota. Healthy diets and regular physical exercise can reduce fat accumulation and enhance gut microbial diversity. Moreover, the utilization of microecologic preparations such as probiotics, prebiotics, and symbiotics plays a significant role in ameliorating childhood obesity by restoring the gut microbiome and improving metabolic dysregulation. Fecal microbiota transplantation refers to the administration of bacterial fluids containing fecal extracts from healthy donors into the recipients’ intestines through oral capsules, nasal feeding, or endoscopy, with the aim of altering chronic low-grade inflammatory responses and establishing an optimal ratio of gut microbiota. Moreover, metabolic surgery is acknowledged as an effective approach for the treatment of severe obesity, currently prevalent surgical procedures encompass vertical sleeve gastrectomy (VSG) and Roux-en-Y gastric bypass (RYGB).

Presently, regulating the composition and function of gut microbiota through dietary intervention, physical exercise, intake of probiotics, prebiotics, and synbiotics, FMT, and metabolic surgery has become a new direction for the prevention and treatment of obesity, as shown in Figure 3.

### Dietary and lifestyle modifications

The role of food in maintaining a healthy gut microbiota is pivotal, given its fundamental significance in human existence. Currently, the primary dietary interventions for managing pediatric obesity involve adjusting macronutrient content, implementing food choice restrictions, and incorporating intermittent energy restriction [180]. Modifications in dietary fatty acid composition can not only impact hepatic lipid accumulation and metabolism but also influence on gut microbiota composition. In mouse models, consumption of a high saturated fatty acid (derived from lard) diet resulted in reduced diversity and richness within the gut microbiota, characterized by a decrease in the abundance of Bacteroidetes and an increase in Firmicutes [181]. Conversely, intake of monounsaturated fatty acids and polyunsaturated fatty acids can enhance gut health by mitigating dysbiosis and metabolic imbalances. Furthermore, the research has demonstrated that a HFD significantly disrupts the circadian rhythm of gut microbiota in rats, leading to decreased concentrations of Bacteroidetes and impaired intestinal barrier function, and subsequent development of obesity. However, time-restricted feeding partially restores ileal microbiota composition (Staphylococcaceae increased), and transcriptome circadian rhythm, while enhancing GLP-1 release, also modulates the bile acid pool in the ileum along with FXR signaling [182]. Previous studies have demonstrated that short-term exposure to a high-nutrient diet before the onset of obesity can lead to impaired insulin sensitivity within the CNS, alterations in the gut microbiota composition, and activation of inflammatory mediators [183]. Therefore, implementing dietary restrictions on daily intake of HFD and high-sugar diets represents an accessible and effective approach for weight reduction in children. However, analysis of the major leukocyte categories in mice fasted for 24 h revealed that intermittent fasting patterns resulted in the migration of monocytes from the bloodstream to the bone marrow within a short timeframe, thereby augmenting the inflammatory response in the body and diminishing host immunity against infection [184]. A wholesome diet should primarily consist of vegetables and grains, aligning with the principles underlying the Mediterranean diet. Currently, the Mediterranean diet exerts a favorable impact on the health of children and adolescents, manifesting anti-inflammatory effects and conferring protection against asthma. Research findings indicate that adherence to the Mediterranean diet is associated with an increased abundance of *Bifidobacterium* sp., elevated concentrations of SCFAs, reduced abundance of *Ruminococcus* sp., and decreased concentrations of C-reactive protein [185]. In comparison with other low-fat dietary patterns, the Mediterranean diet may be more effective in achieving long-term weight loss goals among overweight or obese individuals. Further comprehensive research is required to explore the impact of dietary fat on host metabolism through the gut microbiota and elucidate the underlying mechanisms involved in this pathway.

Trace elements are essential nutrients that play crucial roles in the body’s regulatory, immune, and antioxidant functions, as well as serving as essential components of cofactors for specific enzymes involved in metabolic processes. It has been found that children with obesity exhibit elevated circulating concentrations of copper, decreased plasma concentrations of various trace elements (e.g. cobalt, chromium, manganes, molybdenum, selenium, and zinc), and an accumulation of metals within red blood cells [186]. Previous studies have indicated that metabolic dysfunction associated with obesity and insulin resistance is characterized by disturbances in iron homeostasis and alterations in the composition of gut microbiota [187]. Only 5%–20% of dietary iron is absorbed by the duodenum, leaving ∼80% within the intestinal lumen (primarily colon) for utilization by microbial communities. The availability of iron in the intestinal tract significantly influences gut microbial ecology since nearly all bacteria replication and growth rely on competition for this unabsorbed dietary iron. However, beneficial bacteria like lactobacilli and bifidobacteria require minimal to no iron while not producing any other iron carriers, enrichment of these bacteria in mouse and human microbiota is associated with improved intestinal barrier functions and reduced colonization by intestinal pathogens [188]. Researchers observed that iron supplementation in 6-month-old Kenyan infants stimulated the growth of *Escherichia coli* and *Shigella*, resulting in an increase in the ratio of Enterobacteriaceae to *Lactobacillus*, a decrease in the relative abundance of bifidobacteria, and exacerbation of intestinal inflammation [189]. In contrast, during puberty, children with obesity may exhibit enhanced regulation of oxidative stress and inflammation through buffering mechanisms mediated by micronutrients, which is characterized by elevated concentrations of essential elements (e.g. selenium, manganese, zinc, and molybdenum) involved in antioxidant defenses and metabolic control, as well as reduced concentrations of potentially harmful substances (copper, labile and iron), with the most notable finding being the clearance of unstable iron in pubertal children’s plasma, which is associated with a healthier multielement profile [190]. Micronutrient concentrations in children with obesity may vary depending on poor nutritional habits, and a rational diet can effectively rectify the metabolic disorders associated with micronutrient imbalances. However, further prospective studies with larger sample sizes are needed to explore the causal relationship between changes in micronutrients and the onset of obesity. The response of the human gut microbiome to dietary changes demonstrates a high degree of individual variation. Furthermore, considering the unique characteristics of children, it is imperative to conduct various clinical safety studies on the dietary intervention methods for populations with obesity.

Recently, the regulation of circadian rhythms and metabolism has emerged as a prominent research area. Mounting evidence suggests that disturbances in these rhythms can contribute to various metabolic disorders such as obesity and diabetes. However, the underlying mechanisms remain elusive [191]. The early-life feeding patterns, sleep patterns, and growth trajectories exert long-lasting effects on genetic factors that persist until 3 y of age. A recent study has unveiled that at 6 mo of age, a higher abundance of the *Tissierellales* family in infants who sleep for >13 h while *A muciniphila* and *Bifidobacterium* are found to be more prevalent in infants who sleep for <13 h [192]. Moreover, the findings of a Spanish study revealed disparities in circadian rhythm health among children classified as obese, overweight, and normal weight, with individuals categorized as obese exhibiting poorer circadian rhythm health. Early interventions targeting improvements in circadian rhythm may serve as preventive measures against childhood obesity [193]. Templeman et al. [194] proposed that reducing nocturnal calorie intake and extending overnight fasting periods not only contribute to reduced systemic inflammation but also augment the quality of life. Given the inherent characteristics of meal times and the induction of ketosis during fasting, this approach may not be appropriate for individuals with hypoglycemia or adhering to a ketogenic diet. Additionally, it remains unclear what constitutes an optimal duration of fasting across various age groups in children. Numerous studies have demonstrated that the circadian rhythm exerts regulatory control over organismal metabolism and energy homeostasis by modulating the rhythmic secretion of diverse endocrine hormones and adipokines. A comprehensive investigation into the interaction between the circadian clock and obesity may provide novel insights and therapeutic strategies for preventing and managing obesity, particularly in pediatric populations [191,195].

The regular practice of scientific physical exercise is advantageous in the prevention of obesity. Exercise has been shown to enhance microbiota diversity and increase the abundance of butyric-producing bacteria and SCFAs. In children with obesity, the abundance of *Pasterellaceae*, *Lachnoclostridium*, and *Haemophilus* species was found to decrease in children with obesity after 3 wk of regular training and high-capacity training, while the abundance of *Ruminococcus callidus* increased [196]. The findings of a recent study have demonstrated that a combination of aerobic and resistance training effectively ameliorated HFD-induced microbiota imbalance by upregulating *Parabacteroides*, *Bacteroides*, and *Flavobacterium* species, which are known to be involved in regulating the gut–liver axis and maintaining bile acid homeostasis [197]. Furthermore, the Bacteroidetes can suppress TLR4 and angiotensin-converting enzyme 2 dependent signal transduction, thereby enhancing resistance against proinflammatory cytokines [198]. In the physical structure, exercise has the potential to enhance the structural characteristics of intestinal villi, such as increased thickness, height, and crypt depth, which leads to an improved integrity of the intestinal barrier through tight junctions and mitigates the impact of obesity on gut microbiota [199]. Dietary interventions have the potential to radically shape the composition of healthy and stable gut microbiota, while exercise can contribute to maintaining a balanced gut microbiota. In general, a combination of diet and exercise may offer a more comprehensive approach to managing obesity.

### Microecologic preparations

#### Probiotics

Probiotics usually refer to live nonpathogenic microorganisms used for enhancing the microbial balance within the host [200]. The substantial potential of probiotics in the prevention and treatment of childhood obesity has been demonstrated by numerous studies [201,202]. *Bifidobacterium*, a type of probiotic, can positively influence intestinal barrier function. Solito et al. [203] found that treatment with *Bifidobacterium breve* BR03 and B632 for 8 wk in individuals aged 6–18 y with obesity resulted in weight loss and improved insulin sensitivity. Currently, *Lactobacillus* species demonstrates favorable effects in reducing body fat percentage and maintaining glucose homeostasis [204]. Certain strains of *Lactobacillus* and *A muciniphila* also exhibit potent inhibitory activity against α-glucosidase, thereby impeding the hydrolysis of complex carbohydrates and regulating postprandial hyperglycemia [205]. In infants and children exposed to antibiotics early in life, the supplementation of probiotics may potentially reduce the incidence of childhood obesity by modulating the composition of gut microbiota [206]. Furthermore, researchers have initiated investigations into novel probiotics, such as *Lactobacillus plantarum* 73a (derived from breast milk) and *Bifidobacterium animalis* subsp. lactis INL1 [207]. *Bifidobacterium animalis* subsp. lactis strain BPL1 has demonstrated efficacy in ameliorating central adiposity in adults with simple obesity. In a randomized crossover trial investigating the effects of BPL1 supplementation on children and adolescents with Prader-Willi syndrome, it was observed that BPL1, without altering total adiposity, significantly reduced abdominal fat compared with placebo, leading to improvements in fasting insulin concentrations and insulin sensitivity [208]. Additionally, a study has identified that intake of *Bifidobacterium pseudocatenulatum* CECT7765 improves inflammatory status in children with obesity and insulin resistance [209]. The study conducted by Alisi et al. [210] also demonstrated that supplementation with VSL#3, a probiotic mixture consisting of 8 live bacterial strains) may potentially mitigate obesity. However, a recent study has suggested the opposite, indicating that supplementation with VSL#3 may result in weight gain among individuals already categorized as obese. The study highlighted potential variations in responses to probiotic supplementation based on factors such as age, obesity status, and race/ethnicity [211]. Besides, Zarrati et al. [212] also observed no statistically significant impact on BMI, body fat, and waist-to-hip ratio among obese and overweight individuals following probiotic treatment with *Lactobacillus* and *Bifidobacterium* spp. To date, the majority of human studies investigating probiotics have not reported any discernible antiobesity effects. Therefore, it is necessary for further investigation into reactions associated with probiotics within larger and more diverse study populations.

#### Prebiotics

Prebiotics are a class of nondigestible carbohydrates that can be fermented and used by the gut microorganisms, contributing to the maintenance of a well-balanced gut microbiota. Common prebiotics include oligofructose, galacto-oligosaccharide, inulin, and gynolactos [213]. Probiotics are mainly involved in the regulation of lipid metabolism by stimulating the production of beneficial bacteria and SCFAs, thereby improving the intestinal barrier function and enhancing the body’s resistance against inflammation. Moreover, it can impede the activity of lipogenic enzymes to diminish the synthesis of lipoproteins and triglycerides [214,215]. In light of the favorable benefits observed in adults, inulin-based prebiotics have been extensively used in studies investigating childhood obesity. When compared with a placebo, the administration of oligofructose-enriched inulin selectively modulated the gut microbiota and resulted in a significant reduction in BMI among children with obesity [216]. Furthermore, the incorporation of prebiotics into infant formulas containing galacto-oligosaccharide mixtures can replicate the composition of breast milk and facilitate the proliferation of bifidobacteria in the gastrointestinal tract of infants [217]. Current research on prebiotics for the prevention and treatment of childhood obesity should be further focused on specific aspects, such as the selection of prebiotic types, optimal duration of usage, and appropriate ratios.

#### Synbiotics

The term synbiotic refers to probiotic supplements containing prebiotic components, which potentially yielding more advantageous effects on gut microbiota than that by the ingestion of either probiotics or prebiotics alone. By assigning children with obesity to 3 groups receiving *Lactobacillus casei*, *L casei* in combination with inulin, and *L casei* in combination with fructans, the researchers observed that the symbiotic combination of *L casei* with either inulin or fructans significantly enhanced lipid metabolism and the expression of butyrate and propionate, which are important for gut microbiota regulation [218]. The research on synbiotics, however, remains relatively limited. The approaches such as probiotics and prebiotics can effectively restore the imbalance of gut microbiota in a short period in unhealthy situations. However, it should be noted that once the intervention is discontinued and the patient resumes a normal diet, there may be a gradual disappearance of the dominant gut microbiota obtained. Thus, the combination of multiple approaches such as a well-balanced diet and scientifically designed exercise, along with the usage of probiotics and prebiotics, functions best in maintaining a stable state of gut microbiota and may have cumulative protective effects on regulating the body’s metabolism.

Probiotics, prebiotics, and synbiotics, as an affordable and safe functional food, have emerged as an innovative strategy for preventing or ameliorating diet-induced dysregulation of lipid metabolism in the specific context of rising health care costs for chronic diseases. Currently, probiotics have gained widespread usage in the fields of medicine, health products, and food, a diverse range of probiotic products are available including tablets, powders, capsules, and granules. In many developed countries such as Japan and Europe, pediatricians frequently recommend probiotic medications and dietary supplements for children [200]. The unique nature of child growth and development necessitates the consumption of probiotic preparations in liquid or semiliquid form (e.g. water and milk) for infants and younger children, and it should be aware that the choice of media and temperature may impact the activity of probiotics. Older children can tolerate tablets or capsules well, and chewable probiotic tablets with a pleasant taste are often preferred. Probiotic preparations generally exhibit a high level of safety; however, instances of adverse reactions have also been reported, including systemic infections, gastrointestinal side effects, skin complications, horizontal gene transfer between probiotics and the normal microbiota, and immune system stimulation. The most vulnerable groups include infants, elderly individuals, and those with immunodeficiency resulting from either genetic or acquired diseases [219]. Although probiotic preparations have demonstrated beneficial effects on human health, it is crucial to emphasize the importance of their prudent use, avoiding indiscriminate consumption or prolonged administration without proper evaluation.

### Fecal microbiota transplantation

The FMT is now recognized as a viable therapeutic strategy for childhood obesity. Initially proposed by Eiseman et al. [220] in 1958, FMT refers to the administration of bacterial fluids containing fecal extracts from healthy donors into the recipients’ intestines through oral capsules or nasal feeding, with the aim of altering chronic low-grade inflammatory responses and establishing an optimal ratio of gut microbiota [221]. The efficacy of FMT in treating patients with recurrent and refractory *Clostridium difficile* infections has been demonstrated remarkably in previous studies [222]. Existing experiments in animal and adult models have shown that FMT can alter the composition of gut microbiota, increase gut microbial diversity, improve insulin sensitivity, and reduce oxidative stress-induced tissue damage in individuals with obesity. Interestingly, Wilson et al. [223] discovered that there is competition for bacterial colonization during the utilization of multidonor FMT in the treatment of adolescent obesity. This competition arises due to the dominant donor microbiome possessing a higher *Prevotella*/*Bacteroides* ratio, which enhances its implantation efficiency. Although the study did not observe a reduction in BMI as a result of FMT, preselecting donor microbiomes based on these characteristics may enhance the colonization capacity of donor strains and elicit more significant changes in microbiome functionality. In another investigation, over a period of follow-up, no significant change in the subject’s body weight was observed after the transfer of gut microbiota from healthy individuals to children with obesity via capsules, without altering their diet and lifestyle habits [224]. The role of phage groups in facilitating stability restoration during FMT treatment has been extensively investigated and analyzed, revealing the successful transfer of certain phages from donors to patients and their beneficial impact [225]. However, it is important to emphasize that the utilization of phages or other microbial preparations for modulating gut microbiota should not be regarded as a substitute for conventional disease treatments. Rather, it serves as an adjunctive therapeutic approach with the potential to delay disease progression or act as a preventive measure. This study demonstrated significant changes in the composition and function of gut microbiota among patients following FMT. However, there remains a lack of academic consensus on its potential to ameliorate metabolic disorders or even obesity. This discrepancy may be attributed to factors such as donor selection, sample size and duration of the study, as well as the severity of metabolic disorders exhibited by the subjects.

Compared with probiotic and prebiotic treatments, FMT offers the distinct advantages of a diverse array and substantial quantity of transplanted microbiota and the capacity to optimize the preservation of the original functional bacteria. The study has demonstrated that autologous FMT can restore intestinal microecology disrupted by antibiotics more rapidly and significantly than probiotic supplementation [226]. The innovative clinical treatment approach of FMT, however, necessitates the thorough consideration and discussion of potential risks associated with transplant-related diseases, autoimmune diseases or metabolic disorders, and even cancer. Therefore, it is imperative to further enhance and address the ethical and normative issues surrounding FMT. To date, long-term follow-up studies have demonstrated that FMT exhibits minimal adverse effects, primarily manifesting as mild gastrointestinal discomfort, occasional nausea, and infrequent episodes of vomiting. Clinical practice has shown that the majority of patients can achieve spontaneous recovery or recover after routine management of these adverse effects. In summary, FMT offers a novel perspective for the management of obesity and related metabolic disorders. However, there is a lack of standardized guidelines in several aspects including indications, procedural protocols, amount of fecal fluid transplantation, treatment duration, and safety in long-term application. To address these gaps effectively, it is imperative to conduct high-quality randomized controlled trials and establish long-term follow-up mechanisms for clarification.

### Metabolic surgery

Metabolic surgery may be considered for adolescents with severe obesity (e.g. BMI: 40–35 kg/m^2^ with comorbidities), who experience challenges in glycemic control or experience serious complications despite lifestyle modifications and pharmacologic interventions [227]. Among them, vertical sleeve gastrectomy and Roux-en-Y gastric bypass (RYGB) are widely used. Compared with RYGB, vertical sleeve gastrectomy presents a more favorable option for pediatric patients due to its simplified execution and reduced risk of micronutrient deficiencies. However, concerns persist regarding the safety and long-term efficacy of bariatric surgery [228]. A recent study revealed a potential causal association between *B thetaiotaomicron* in the intestinal tract and obesity in individuals, as shown by the decrease in *B thetaiotaomicron* within the intestinal tract of patients with obesity who underwent RYGB, which exhibited a significant elevation 3 mo after the bariatric surgery. Simultaneously, serum glutamate concentrations also decreased significantly to concentrations comparable with those of people with normal body weights after the surgery [140]. The findings imply that *B thetaiotaomicron* could serve as a promising target for the development of probiotic-based weight loss drugs or functional foods.

Additionally, significant associations have been observed between enhanced dietary behavior resulting from bariatric surgery and alterations in the functionality of the putamen within the basal nucleus, a pivotal reward-related brain region, and associated with modifications in the composition of the gut microbiome. The RYGB and laparoscopic sleeve gastroplasty surgeries induce alterations in brain function within the reward center associated with obesity, augmenting the activity of GLP-1 and regulatory peptides in the CNS, thereby modulating food preference and mitigating food addiction, ultimately resulting in effective weight loss [229,230]. After transplanting the fecal microbiota of the same patient before and after bariatric surgery into the mice gut, it has been observed that the gut microbiota after bariatric surgery did not affect insulin clearance and insulin resistance in mice while improving blood glucose concentrations by reducing intestinal glucose absorption mediated by sodium-glucose cotransporter protein 1 [231]. In light of these findings, further investigation is warranted to develop probiotics and prebiotics capable of emulating the microbiologic effects of bariatric surgery, reducing intestinal glucose absorption, and overcoming the limitations associated with surgical weight loss.

## Summary

Currently, the gut microbiota has been acknowledged as an additional contributing factor favoring fat storage, weight gain, and insulin resistance. Disturbances in the gut microbiota may contribute to the development of childhood obesity through mechanisms such as disturbed energy metabolism, endocrine disruption, and inflammation. The management of pediatric obesity requires a variety of strategies encompassing lifestyle management, pharmacologic treatment, metabolic surgery, and so on. The crucial role played by certain gut microbiota in childhood obesity renders it a promising target for novel antiobesity therapies. However, the limited amount of human research along with interspecies variations, interindividual differences, and metabolic deviations impede translating relevant findings from rodent models into humans effectively. Moreover, the utility of microbiome for obesity management is still restricted to research settings, the limited application of microbiome-based interventions for obesity treatment in clinical settings leaves the efficacy of microbiota therapies uncertain. In addition, further optimization is required for gut microbial therapy as a novel technology to be applied in the management of childhood obesity, due to the absence of standardized clinical practice protocols currently. More large-scale, high-quality prospective studies are necessary to investigate the relationship between the gut microbiota and childhood obesity and to explore the effects of diverse microbial therapeutic regimens on ameliorating obesity in children. Regardless of how the core bacteria of a healthy gut are defined and the potential variations in identification results among population studies, it is indisputable that understanding the common and dominant core bacteria is pivotal for maintaining gut homeostasis and overall health. By establishing a robust foundation of microbial markers for early detection of obesity and addressing existing gaps in our understanding of the relationship between gut microbiota and childhood obesity, valuable insights can be provided for effective management strategies targeting prevalent childhood obesity.

## Author contributions

The authors’ responsibilities were as follows—YL, BT: developed the concept for the manuscript; YL, DL, BT: searched the literature, designed the figure, and wrote the manuscript; ML, BT: provided substantial contribution to the structuring and content design of this review, as well as critical revision of the article; BT: had primary responsibility for final content; and all authors: read and approved the final manuscript.

## Funding

This work was supported by the Special Foundation of Sichuan Academy of Medical Sciences and Sichuan Provincial People’s Hospital (No. 2021ZX04) and Open Fund of the Key Laboratory of Birth Defects and Related Diseases of Women and Children, Ministry of Education, West China Second University Hospital, Sichuan University (grant number 2022KF03).

## Data availability

No data sets were generated or analyzed during this study.

## Conflict of interest

The authors report no conflicts of interest.
